# Versatile Application of TiO_2_@PDA Modified Filter Paper for Oily Wastewater Treatment

**DOI:** 10.3390/molecules28237903

**Published:** 2023-12-02

**Authors:** Chang-Hua Zhao, Yu-Ping Zhang, Li Wan, Xin-Xin Chen, Pei Yuan, Ling-Bo Qu

**Affiliations:** 1 College of Chemistry, Zhengzhou University, Zhengzhou 450001, Chinaqulingbo@zzu.edu.cn (L.-B.Q.); 2 College of Chemistry and Materials Engineering, Hunan University of Arts and Science, Changde 415000, China; 3 College of Chemistry and Chemical Engineering, Henan Institute of Science and Technology, Xinxiang 453003, China

**Keywords:** TiO_2_@PDA, filter paper, oil/water separation, emulsion separation, purification

## Abstract

Although membrane separation technology has been widely used in the treatment of oily wastewater, the complexity and high cost of the membrane preparation, as well as its poor stability, limit its further development. In this study, via the vacuum-assisted suction filtration method, polydopamine (PDA)-coated TiO_2_ nanoparticles were tightly attached and embedded on both sides of laboratory filter paper (FP). The resultant FP possessed the typical wettability of high hydrophilicity in the air with the water contact angle (WCA) of 28°, superoleophilicity with the oil contact angle (OCA) close to 0°, underwater superoleophobicity with the underwater OCA greater than 150°, and superhydrophobicity under the water with the underoil WCA over 150° for five kinds of organic solvents (carbon tetrachloride, toluene, n-hexane, n-octane, and iso-octane). The separation efficiency of immiscible oil/water, oil-in-water, and water-in-oil emulsions using the modified FP is higher than 99%. After 17 cycles of emulsion separation, a high separation efficiency of 99% was still maintained for the FP, along with good chemical and mechanical stability. In addition, successful separation and purification were also realized for the oil-in-water emulsion that contained the methylene blue (MB) dye, along with the complete degradation of MB in an aqueous solution under UV irradiation.

## 1. Introduction

The pollution caused by oil spills in industrial production and the discharge of oily wastewater into everyday life has become a greater threat to environmental safety and human health as modern society has become more industrialized and urbanized [1,2,3]. Immiscible oil/water mixtures can be treated with conventional oil/water separation methods, including skimming, gravity deposition, air flotation, centrifugal separation, electrochemistry, adsorption, etc. [4]. However, due to the small size of oil/water droplets, surfactant-stabilized oil/water emulsions cannot be treated with these traditional methods [5,6]. Through the synergistic effect of introducing hydrophilic substances into the membranes and constructing micro- and nanostructures on the membrane surface, researchers have prepared membranes with superamphiphilicity in the air and superamphiphobicity (i.e., oleophobicity under water and hydrophobicity under oil), which are capable of separating oil-in-water (O/W) emulsions and water-in-oil (W/O) emulsions by pre-infiltration. On the other hand, most membranes developed so far could only separate emulsions into the oil and water phases, not the contaminants in the water phase. Serious oil fouling, instability under harsh conditions, and abrasion wear during cycling also limit their application [7,8,9,10]. Nanomaterials with photocatalytic degradation ability are usually selected to modify the separation membrane in order to change or further improve the antifouling performance of the membrane under light irradiation. At present, the development of novel hybrid adsorbent/photocatalytic nanomaterials for separation and purification technologies represents one of the most attractive opportunities in the field of water treatment [11,12,13].

Various membrane materials, such as metal mesh [14], ceramic membranes [15], paper-based materials [16], and polymer membranes [17], have been tried for oil/water and emulsion separation. However, metal mesh has a large pore size and is poor for stabilizing emulsion separation; ceramic membranes are prone to scaling and clogging; and most polymer membranes have low fluxes and poor mechanical properties. Commercially available laboratory FP is an economical, renewable, bio-degradable material containing tiny pore sizes that has a wide range of applications for oil/water and emulsion separation [18,19]. Meng developed a superhydrophobic cellulose–chitosan composite aerogel through electrostatic interaction and ion exchange. The fabricated novel materials were successfully used to separate free oil and surfactant-stabilized W/O emulsions [20]. Xu et al. reported a modified cellulose membrane for effective O/W emulsion separation. The cellulose-polyvinyl alcohol (PVA) membrane fabricated by a universal method of hydroxyl oxidation demonstrated superhydrophilicity, superoleophilicity in air, and superoleophobicity under various pH value solutions. The resultant membrane, with good chemical and mechanical stability, can dispose of many types of O/W emulsions by gravity [21]. Zhao et al. proposed a facile strategy to prepare a kind of water-repellent paper using a self-crosslinkable polyelectrolyte (PCMVIm). Paper sheets incorporated with 0.6 wt% PCMVIm were rendered water-proof and acid-resistant, exhibiting a wet strength that is six times higher than pristine paper without PCMVIm. The modified paper sheets with both high strength and changeable surface properties exhibited high permeability, selectivity, and stability in separating water/oil emulsions [22]. Ren et al. prepared a pH-responsive superhydrophobic coating composed of TiO_2_, chitosan, and stearic acid on three different material surfaces (cotton fabric, sponge, and FP). The modified materials could effectively separate oil/water mixtures and emulsions [23]. Superoleophilic paper was initially fabricated through in-situ growing Cu metal-organic framework nanoparticles and simple layer-by-layer self-assembly. The subsequent superhydrophobic modification was carried out with a polydimethylsiloxane treatment. The resultant paper demonstrated excellent separation performance for oil/water mixtures and emulsions, along with good mechanical and chemical durability [24]. A durable superhydrophobic ZnO coating on a FP was successfully fabricated by four cycles of ZnO microparticle deposition, followed by the chemical modification of stearic acid. The resultant paper exhibited superhydrophobic properties and excellent long-term stability [25]. However, none of the modified filtration membranes in the above work were mentioned for the removal of contaminants, and only a single removal of O/W or W/O emulsion was possible. Therefore, it is valuable to develop a simple, versatile, and green method for preparing multifunctional membranes that can effectively separate O/W and W/O emulsions, along with removing contaminants at the same time.

Dopamine is a neurotransmitter containing catechol and primary amine functional groups. It can be oxidized under aerobic alkaline conditions and self-polymerized to form PDA, which adheres to a variety of substrates through covalent reactions. Furthermore, PDA is an ideal molecular binder that can greatly improve the stability of the modified film [26,27]. Since being inspired by the secretion of adhesion proteins by mussels, PDA has been used by many researchers as a surface modifier, intermediate layer, skin layer, etc. [28,29]. At present, there are few reports on the use of PDA-modified TiO_2_ in oil/water separation.

Herein, a lab FP was successfully modified via a one-step suction filtration process without any hydrophobic modification. The as-prepared paper embedded with polydopamine-coated TiO_2_ (TiO_2_@PDA) nanoparticles presented highly hydrophilic and superoleophilic properties. The interaction force between the substrate surface and coating was improved with the help of adhesive PDA and suction filtration back and forth. The resultant highly hydrophilic nanoparticles were strongly embedded on the substrate surface for recyclable usage. It enables on-demand separation of immiscible light oil/water mixtures as well as heavy oil/water mixtures. Moreover, the paper was successfully used to separate surfactant-stabilized emulsions and exhibited high separation efficiency for both O/W and W/O emulsions (over 99.0%). Meanwhile, the O/W emulsion containing MB dye was successfully separated and purified, while the MB in the emulsion was utterly adsorbed on the FP simultaneously. Meanwhile, highly efficient removal of MB dye was carried out by composite FP based on the synergistic effect of adsorption and photocatalysis by UV irradiation. To the best of our knowledge, this kind of multifunctional FP, which has the ability to separate oil/water mixtures and emulsions and selectively adsorb and degrade organic pollutants, was less reported in previous reports. In addition, both nanocomposites of TiO_2_ with different size and weight percentages and ZnO exhibited highly amphiphilicity in the air and superamphiphobicity under liquid. Furthermore, considering its good durability, reusability, and universality, we believe that this specially modified FP will inspire multifunctional applications for the treatment of oily wastewater in the future.

## 2. Results

### 2.1. Morphology Characterization

**Figure 1:**
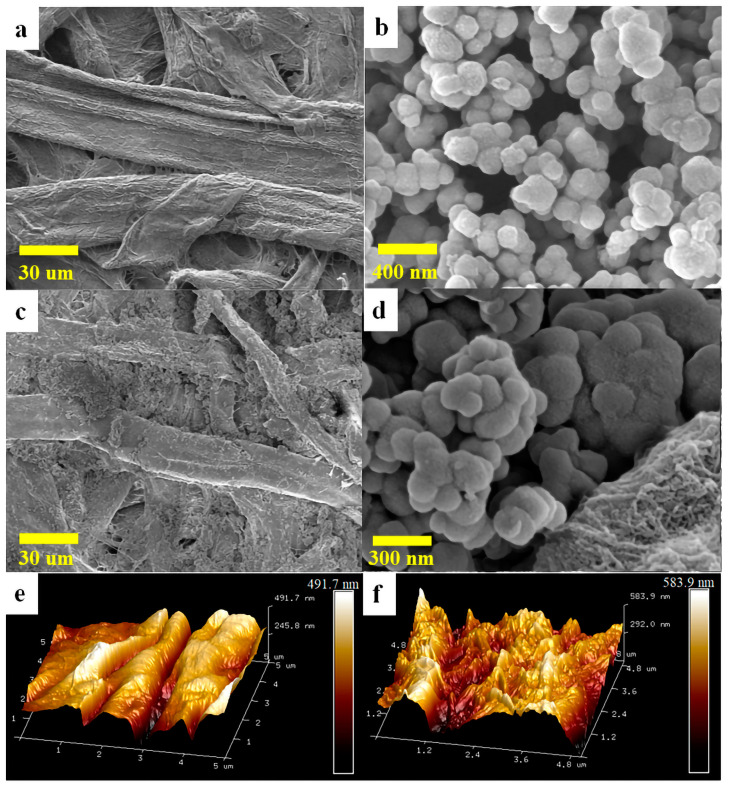
SEM images of original FP (**a**) 100 nm TiO_2_@PDA (**b**) modified FP magnification at 600× (**c**) 50,000× (**d**). AFM scanning result of original FP (**e**) modified FP (**f**).

The scanning electron micrograph (SEM) of FP before and after modification are shown in Figure 1a–d. The fiber surface of the pristine FP was smooth, and the prepared TiO_2_@PDA nanocomposites were uniformly spherical with a particle size of 80 to 180 nm (Figure 1b). The surfaces and pores of the FP were filled with TiO_2_@PDA nanoparticles after vacuum-assisted suction filtration, which reduced the pore size of the FP (Figure 1c,d). The mass of modified FP increased by 6–7% due to the attached and embedded TiO_2_@PDA nanoparticles, compared with the pristine FP. Moreover, the modification of TiO_2_@PDA nanoparticles altered the surface wettability of the FP and increased the surface roughness. The hierarchical micro-nanostructure was thus formed, which was conducive to the separation and purification processes.

The three-dimensional surface topography of the pristine and modified FPs was characterized by an atomic force microscope (AFM, Figure 1e,f). In order to further quantify the changes in the membrane surface, the AFM images were processed and analyzed by the NanoScope Analysis 1.7 software, and the roughness of the modified FP increased from 52.0 nm to 67.5 nm. It illustrated that the deposition of nanoparticles resulted in an increase in the roughness of the material surface.

**Figure 2:**
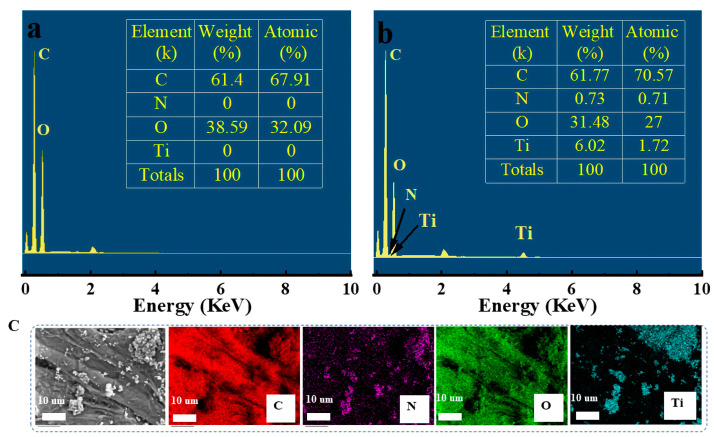
EDS and elemental composition of original FP (**a**); EDS and elemental composition of TiO_2_@PDA@FP (**b**); and elemental distribution on the surface of TiO_2_@PDA@FP (**c**).

The energy-dispersive X-ray spectroscopy (EDS) in Figure 2a,b showed that the atomic percentages of Ti and N elements in the modified FP increased by 1.72% and 0.71%, respectively, compared with the pristine FP, confirming the composition of TiO_2_@PDA nanoparticles. The elemental area distribution map of TiO_2_@PDA@FP indicated that the relevant C, N, O, and Ti elements were distributed on the surface of the FP (Figure 2c).

### 2.2. Wettability

**Figure 3:**
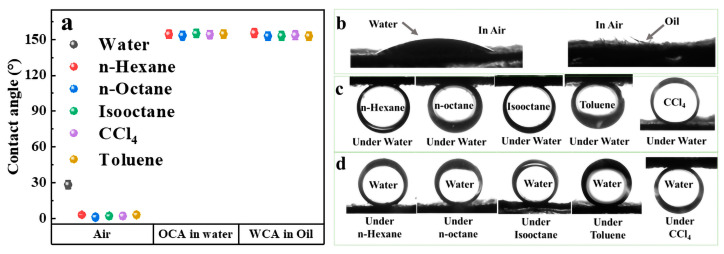
Contact angle sizes (**a**) and corresponding contact angle photos (**b**–**d**) of TiO_2_@PDA@FP in different environments.

The key factor for oil/water and emulsion separation is the specific surface wettability of the material, which depends mainly on the chemical composition and rough structure of the material surface [30,31,32,33]. The size of the contact angle was used to characterize the surface wettability of the FP material, and the WCA, OCA, underwater oil contact angle (UWOCA), and underoil water contact angle (UOWCA) were measured for the filter membrane, and the results are shown in Figure 3. When in air, the oil droplets can spread rapidly on the membrane with an OCA close to 0°, while the water droplets spread slower compared with the oil droplets and exhibited a WCA of 28° on the FP. In the environment under water or oil, the oil droplet or water droplet were in a stable spherical shape to seat on the membrane with both the UWOCA and the UOWCA greater than 150°, respectively (Figure 3a). The typical surface wettability of highly hydrophilic and super-oleophilic in air, super-oleophobic underwater, and super-hydrophobic under oil assured successful applications for oil/water and emulsion separation.

### 2.3. Oil/Water Separation

**Figure 4:**
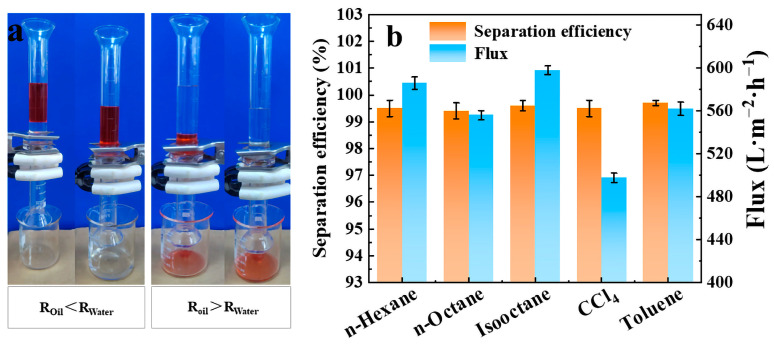
TiO_2_@PDA@FP oil/water separation diagram (**a**) light oil/water separation (**left**), heavy oil/water separation (**right**), separation efficiency, and flux (**b**).

The separation ability of TiO_2_@PDA@FP was evaluated by a series of oil/water separation experiments. When the light oil/water mixture (oil dyed red) was exposed to TiO_2_@PDA@FP, the water wetted the FP first, and the aqueous phase then permeated through the FP while the oil phase was rejected above the FP (Figure 4a, left). When the heavy oil/water mixture (oil dyed red) came into contact with TiO_2_@PDA@FP, the oil wetted the FP first, and the oil phase penetrated while the water phase was blocked above the FP (Figure 4a, right). The separation flux and separation efficiency of TiO_2_@PDA@FP were calculated and analyzed as in Figure 4b, and the permeation flux of each oil/water system was above 480 L∙m^−2^∙h^−1^, and the separation efficiencies of the five oil/water mixtures were above 99%.

### 2.4. Emulsion Separation

**Figure 5:**
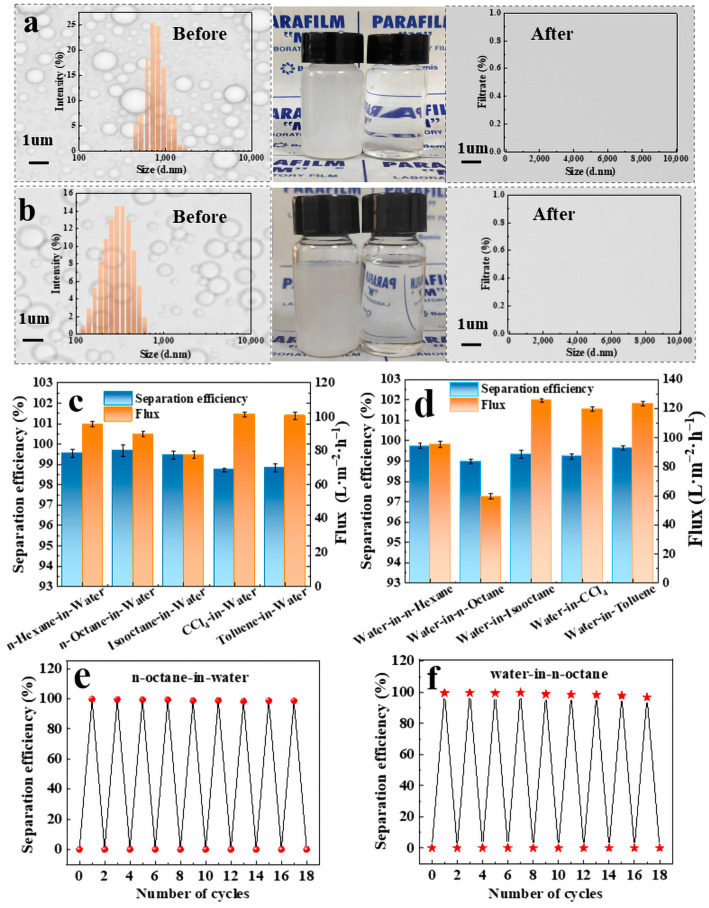
Microscope images of n-octane-in-water emulsion before and after separation (inset shows particle size distribution) (**a**); microscope images of water-in-n-octane emulsion before and after separation (inset shows particle size distribution) (**b**); separation performance of TiO_2_@PDA@FP for different O/W (**c**) and W/O (**d**) emulsions; separation efficiency of TiO_2_@PDA@FP recycled 17 times for separation of n-octane-in-water (**e**) and water-in-n-octane (**f**).

The modified FP with good wettability enables the complete separation of O/W and W/O emulsions by gravity alone. Figure 5a,b show the digital photographs of n-octane-in-water and water-in-isooctane emulsions before and after separation, as well as microscope images, from which it could be seen that before separation, the emulsions were milky white in color, and the microscope images showed different sizes of oil droplets or water droplets. After separation, the filtrate was clear and transparent, and no spherical droplets of different sizes appeared in the microscope images, which indicated that the emulsions had been successfully separated. The emulsion has also been successfully separated, as evidenced by the particle size distribution before and after separation (inset of Figure 5a,b). For the O/W and W/O emulsions before separation, the sizes of oil and water droplets ranged from 458 to 1718 nm and 122 to 615 nm, respectively. The oil droplets in water and water droplets in oil were not detected after separation, which demonstrated emulsion separation was successfully carried out using the modified FP.

The separation efficiency and flux of different O/W and W/O emulsions are shown in Figure 5c,d. It can be seen that the separation efficiency of the emulsions was up to 99%, and the flux of O/W was 77–102 L∙m^−2^∙h^−1^, while W/O showed a slight increase in flux compared with O/W, which was 60–124 L∙m^−2^∙h^−1^. The emulsion separation mechanism can be understood as follows: when the O/W emulsion contacted the surface of the filter membrane, the water first contacted the membrane surface and quickly formed a water film on the membrane surface, which prevented the oil droplets in the emulsion from passing through the FP to enter the filtrate. When poured into the W/O emulsion, the oil quickly formed a layer of oil film on the surface of the membrane, which prevented the water droplets from passing through and realized its ability to break the emulsion. In order to test the recycling performance of the modified FP, both emulsions, such as n-octane/water and water/n-octane, were selected for investigation with a total volume of 15 mL for both emulsions, respectively, and the efficiency of the emulsion separation was still as high as 99% after 17 times cycle usage (Figure 5e,f). It should be noted that the modified FP was not dried and cleaned with ethanol and distillation water during the reuse cycles, which indicated that the as-prepared FP was durable. Chemical stability was also investigated using different corrosives (see Figure S1). The as-prepared FP substrates were immersed in 1 M HCl, NaOH, and NaCl for about 10 h, respectively. Separation efficiency was still higher than 98% for O/W and 85% for W/O, although the relative UWOCA and UOWCA decreased slightly in the range of 142–146° and 132–140°, respectively. Moreover, ultrasonic testing was used for the investigation of mechanic stability (see Figure S2). After ultrasonic vibration over 50 min, separation efficiency was still over 98% for both emulsions, although the relative UWOCA and UOWCA decreased slightly in the range of 138–151°.

### 2.5. In Situ Separation and Purification of Contaminated Emulsion

**Figure 6:**
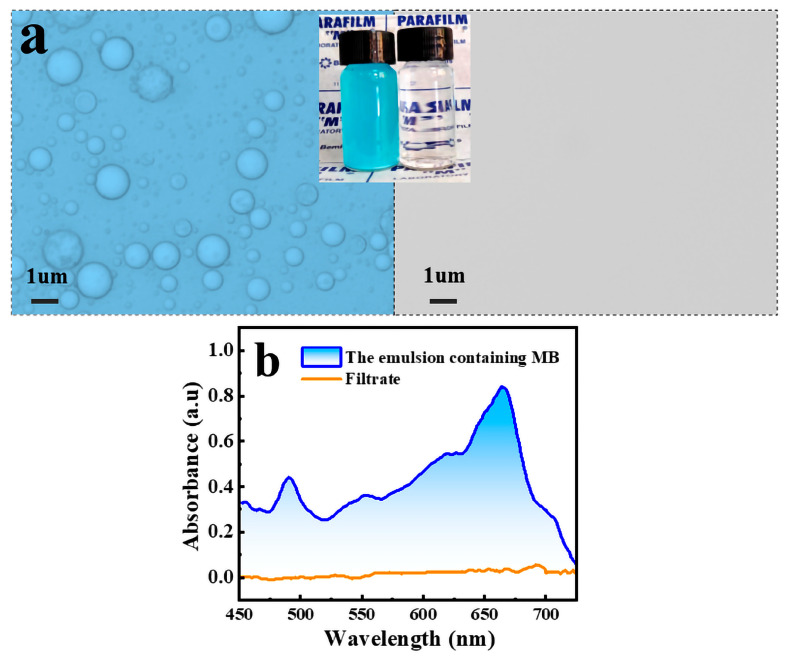
Microscope images of n-octane-in-water emulsion containing 5 mg/L MB dye before and after separation (inset shows photographs of emulsion before and after separation) (**a**). UV-VIS spectra of the feed emulsion (blue line) and the collected solution (orange line) (**b**).

The modified FP was also attempted to adsorb the 5 mg/L MB dye in the n-octane/water emulsion. After filtration, the water in the filtrate became clear and colorless. Figure 6a shows that successful emulsion separation was carried out using the as-prepared FP. The UV scanning curves in Figure 6b illustrated the change of MB concentration before and after filtration, and the characteristic absorption peak of MB at 664 nm in the filtrate completely disappeared, and the used FP turned blue, which indicated that it adsorbed the MB dye. It is evident that the prepared FP can realize separation and purification in one step. The presence of a large number of phenolic compounds on the PDA resulted in the surface being negatively charged, which can realize the adsorption of MB in the emulsion through electrostatic interactions with cationic dyes [34]. In addition, MB is an ideal planar molecule with an aromatic backbone, and PDA also contains abundant aromatic rings, which may undergo π-π stacking interactions with MB [35,36].

### 2.6. Degradation of Soluble Contaminant under UV Irradiation

**Figure 7:**
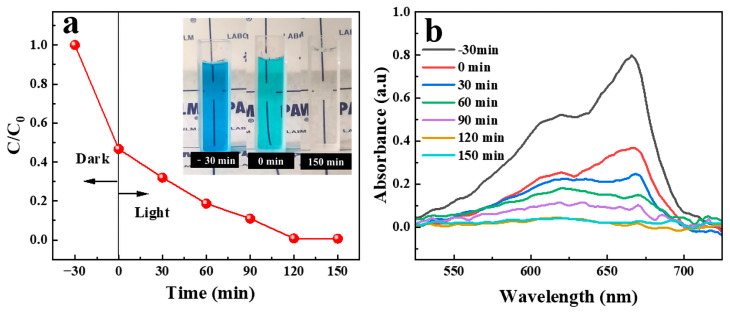
Removal efficiency of MB solution with different UV irradiation times (**a**); absorbance curves of MB at different UV irradiation times (The red line indicates that the reaction was carried out under dark conditions for 30 min before the UV lamp was turned on) (**b**).

It is well known that TiO_2_ converts MB to H_2_O and CO_2_ under UV irradiation with good photodegradation efficiency [37,38]. The surface of modified FP contained TiO_2_ nanoparticles; therefore, the removal of MB dye can be realized under UV irradiation. With the increase in irradiation time, the MB peak at a wavelength of 664 nm gradually decreased, and it completely disappeared when the irradiation time was over 120 min (Figure 7), which proved that the MB was almost completely degraded. The images of the MB solutions inserted in Figure 7a were the initial MB solution, the solution after being absorbed for 30 min, and UV irradiation for 150 min, respectively. The degradation mechanism is that when TiO_2_ is irradiated under UV-VIS at a wavelength lower than 385 nm, the electrons will be excited from the valence band to the conduction band of TiO_2_, generating electron-hole pairs. The photogenerated electrons are easy to capture by the oxygen in the water to generate the superoxide radicals (•O_2_^−^), and the holes are easy to capture by the OH^−^ on the surface of TiO_2_. Among them, -O_2_^−^ and •OH radicals can oxidize MB and generate small molecules or even CO_2_ and H_2_O [39,40,41].

### 2.7. Generalization of the Developed Method

**Figure 8:**
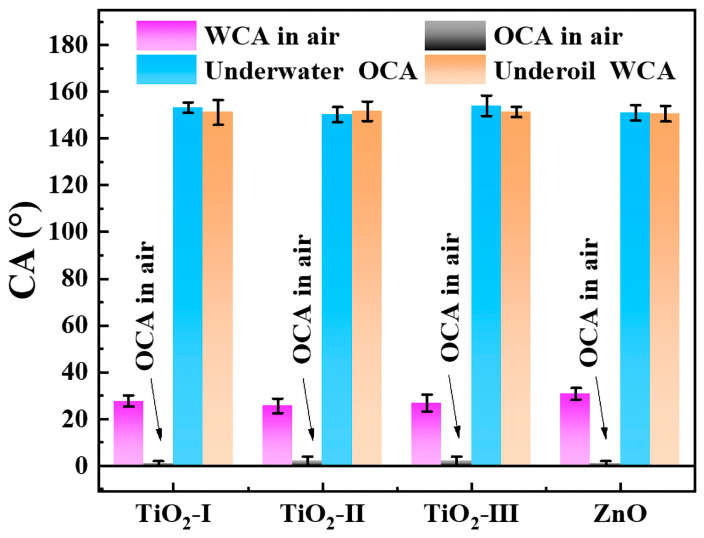
Comparative surface wettability of the as-prepared FPs in the air for water, n-octane, underwater for n-octane, and underoil for water.

In order to investigate the generalizability of the preparation method, TiO_2_ nanoparticles with different sizes and weight percentages were selected for the comparative investigation of wettability along with ZnO nanoparticles. The results suggested that all four FPs attached by PDA-encapsulated nanoparticles, such as TiO_2_-I (25 nm, 100%), TiO_2_-Ⅱ (25 nm, 50%; 100 nm, 50%), TiO_2_-III (25 nm, 25%; 100 nm, 75%), and ZnO nanoparticles (20–40 nm), possessed similar wettability in different environments. Four kinds of modified FPs exhibited similarly hydrophilic (WCA of 25.7°–30.8°) and super-oleophilic (OCA close to 0°) properties in the air, super-oleophobic underwater (UWOCA > 150°), and super-hydrophobic under oil (UOWCA > 150°) (Figure 8). It demonstrated that the developed strategy of “several birds with one stone” was facile and convenient for the fabrication of multifunctional membranes [42,43,44].

## 3. Experimental Section

### 3.1. Materials

TiO_2_ (100 nm) was purchased from Shanghai MackL in Biochemical Co., Shanghai, China. TiO_2_ (25 nm, 100 nm), dopamine hydrochloride (DA, 98%), tris (hydroxymethyl) aminomethane (Tris, ≥99%), Span 80, n-octane (GC, >99%), iso-octane (GC, >99%), n-hexane (GC, >99%), NaCl (AR, 99.5%), ZnO (20–40 nm, 99.9%), NaOH (AR, 96%), and methylene blue trihydrate (MB) were purchased from Aladdin Biochemical Co. CCl_4_ (98%) was purchased from Shanghai Myriad, and toluene (AR) was purchased from Tianjin Fuyu Fine Chemical Co., Tianjin, China. Middle qualitative FP (Φ = 7 cm) was supplied by Jiangsu Taizhou Auke Filter Paper Factory.

### 3.2. Preparation of Multifunctional FP

**Figure 9:**
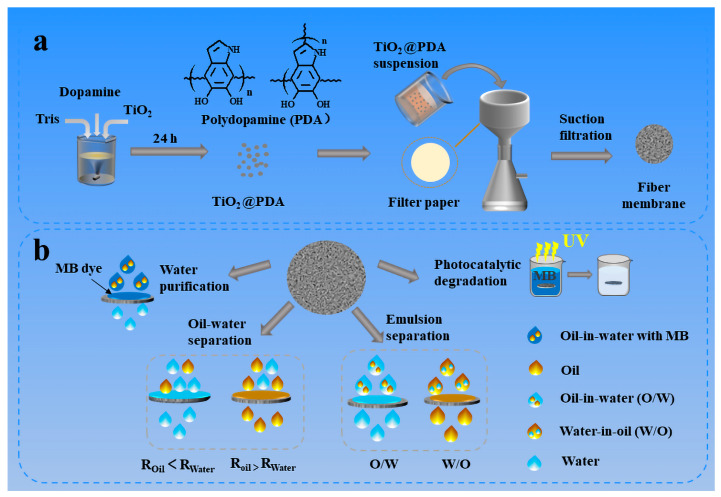
Schematic diagram of the preparation process of TiO_2_@PDA@FP (**a**). Application of composite FP in oil/water separation, emulsion separation, water purification, and photodegradation (**b**).

As illustrated in Figure 9a, first, 0.5 g TiO_2_ nanoparticles were ultrasonically dispersed in 100 mL of dopamine solution, in which DA was 0.2 g and Tris was 0.1 mol·L^−1^ (pH = 8.5), magnetically stirred for 24 h, washed with deionization (DI) water several times, and dried to obtain TiO_2_@PDA nanoparticles. Subsequently, 0.1 g of TiO_2_@PDA nanoparticles were placed in 100 mL of DI water and ultrasonically dispersed for 20 min to obtain 1 g/L of TiO_2_@PDA suspension. Blank FP was dried at 60 °C for 1 h in order to remove water and weigh its mass m_1_, then placed in a Brinell’s funnel, turned on the vacuum pump, moistened with DI water, and then evenly and slowly poured into 10 mL of TiO_2_@PDA suspension. The pressure value of the vacuum pump was 0.03–0.05 MPa, and then when the suspension was filtered completely, the vacuum pump was turned off, and the FP was flipped over, and then the pumping and filtration process was repeated twice, which was dried in an oven at 60 °C and weighed the mass m_2_. Finally, the TiO_2_@PDA@FP was used for multifunctional purposes, including oil/water separation, emulsion separation, in-situ water purification, and photogradation of organic dye, which are shown in Figure 9b in detail.

### 3.3. Oil/Water Separation

The oil/water separation performance of TiO_2_@PDA@FP composites was measured by gravity-driven oil/water mixture separation experiments. The oil/water mixture was prepared by mixing DI water with Sudan III-stained oil (CCl_4_, toluene, n-hexane, n-octane, and iso-octane) at a volume ratio of 1:1. A 1/4 sheet of TiO_2_@PDA@FP was secured between two customized glass tubes, and the oil/water mixture was slowly poured through the top end of the glass tubes. A gravity-driven separation process occurs as the water or oil rapidly passes through the FP. The filtrate was collected, and the water (oil) flux was calculated based on the permeate volume per unit of time. The equation is [27]:

Flux = V/St(1)

where V, S, and t are the filtrate volume (L), effective separation area (m^2^), and permeation time (h), respectively. The separation efficiency (R_1_) was calculated as [16]:

R_1_ = m/m_0_ (2)

where m_0_ and m are the masses of the water before and after the separation process.

### 3.4. Emulsion Separation

For the surfactant-stabilized O/W emulsions, 1 mL of oil phase (CCl_4_, toluene, n-hexane, n-octane, and iso-octane) and 99 mL of DI water were mixed with the addition of 0.1 g of span 80 as an emulsifier. The mixture was stirred for 5 min at 5000 r/min in a high-speed homogenizer to obtain a stable emulsion. Similarly, five types of surfactant-stabilized W/O emulsions were prepared using the same method but with a water-oil ratio of 99:1 (W/O) by volume. The different emulsions were poured from the glass tube above the fixture, and a clean beaker was placed below to collect the filtrate. The separation flux of the FP was calculated according to Equation (1), and the separation efficiency (R_2_) was calculated according to the following equation [45]:

R_2_ = (1 − C/C_0_) × 100% (3)

where C and C_0_ are the water (oil) concentrations in the filtrate and the original emulsion, respectively, as determined by a moisture meter and a UV-vis spectrophotometer.

### 3.5. Water Purification

A typical n-octane-in-water emulsion with a 5 mg/L aqueous MB solution was selected for the investigation of purification performance for the modified FP. After it was poured into the upper glass tube of the experimental set-up, the concentration of MB before and after separation was determined by UV-Vis NIR spectroscopic scanning.

### 3.6. Photodegradation

A 100 mL beaker was filled with 30 mL of 5 mg/L MB solution and 1/4 sheet of FP. After the beaker was placed in a dark environment under magnetic stirring for 30 min, the adsorption-desorption equilibrium was reached. The beaker was then transferred to a UV cross-linker containing five 254 nm lamps with a power of 10 W for the photoreaction, with a distance of 8 cm between the liquid surface and the lamp, and 3 mL of solution was taken out for the measurement every 30 min. The photodegradation performance of the FP was evaluated by detecting the absorbance of MB dye at 664 nm. The photocatalytic degradation efficiency (θ) of MB was calculated by the following equation [46]:

θ = (1 − A_t_/A_0_) × 100% (4)

where A_t_ is the absorbance of MB solution at time t and A_0_ is the initial absorbance of MB solution.

### 3.7. Characterizations

SZ03-Ⅱ Ultraviolet Crosslinker (Topher Electro-Mechanical Technology Co., Ltd., Shanghai, China), PE 750s UV-VIS-NIR spectrophotometer (Platinum Elmer Instruments Co., Ltd., Shanghai, China) to test the concentration of oil and MB, F-020 Fuyang brand ultrasonic cleaner (Shenzhen Fuyang Science and Technology Group Co., Ltd., Shenzhen, China), SEM and EDS (Zeiss Sigma 300 + Oxford Spectroscopy), AFM (Bruker Demension Icon, Bruker (Beijing) Technology Co., Beijing, China), NOVEL NE 620 Optical Microscope (Ningbo Yongxin Optical Co., Ltd., Ningbo, China), TST-300H Optical Contact Angle Measuring Instrument (Testin Test Equipment Co., Ltd., Shenzhen, China), Moisture content was determined by FK-WS1 Karl Fischer Moisture Titrator (Shandong Fangke Instrument Co., Ltd., Weifang, China), the particle size distribution of the emulsions was determined using a Zetasizer Nano ZSE laser particle size-zeta potential analyzer (Malvern Instruments Ltd., Shanghai, China).

## 4. Conclusions

In summary, this work provides a simple, facile, and low-cost method to fabricate the typical superwettable paper for oil/water separation and oily wastewater treatment. The experiments began with the encapsulation of titanium dioxide nanoparticles by dopamine, followed by vacuum-assisted suction filtration of the prepared nanoparticles onto the surface or inside the laboratory FP. The resultant FP has typical wettability properties: highly hydrophilic and super oleophilic in air; super oleophobic under water; and super hydrophobic under oil. Satisfactory separation of immiscible water/light oil, heavy oil/water, O/W, and W/O emulsions was achieved using modified FP. After 17 cycle times of emulsion separations, the separation efficiency of the FP was still as high as 99%, with good chemical and mechanical stability. In addition, the modified FP allowed for the separation and purification of O/W emulsions containing methylene blue (MB) dye while achieving complete UV degradation of MB. This work developed a facile and ingenious strategy of “killing several birds with one stone” to modify laboratory FPs for multi-functional application.

## Data Availability

The data presented in this study are available on request from the corresponding author.

## Supplementary Materials

The following supporting information can be downloaded at: https://www.mdpi.com/article/10.3390/molecules28237903/s1, Figure S1: (a) Separation efficiency and the changes of UWOCA for O/W emulsion; (b) Separation efficiency and the changes of UOWCA for W/O emulsion (Oil is n-octane); Figure S2: (a) Separation efficiency and the changes of UWOCA for O/W emulsion; (b) Separation efficiency and the changes of UOWCA for W/O emulsion (Oil is n-octane).
