# Supplementary material for: Versatile Application of TiO2@PDA Modified Filter Paper for Oily Wastewater Treatment
Source: Molecules. 2023 Dec 2;28(23):7903. doi: 10.3390/molecules28237903 (PMC10708479; doi:10.3390/molecules28237903)
Supplement: Supplementary file 1 [file molecules-28-07903-s001.zip › molecules-2751300-supplementary.pdf]

Versatile application of TiO_2@PDA modified filter paper for oily wastewater treatment

Chang-Hua Zhao [1], Yu-Ping Zhang [2,*], Li Wan[2], Xin-Xin Chen[3], Pei Yuan[2] and Ling-Bo Qu[1]

1 College of Chemistry, Zhengzhou University, Zhengzhou 450001, China
2 College of Chemistry and Materials Engineering, Hunan University of Arts and Science, Changde, 415000, China
3 College of Chemistry and Chemical Engineering, Henan Institute of Science and Technology, Xinxiang, 453003, China
* Correspondence: beijing2008zyp@163.com

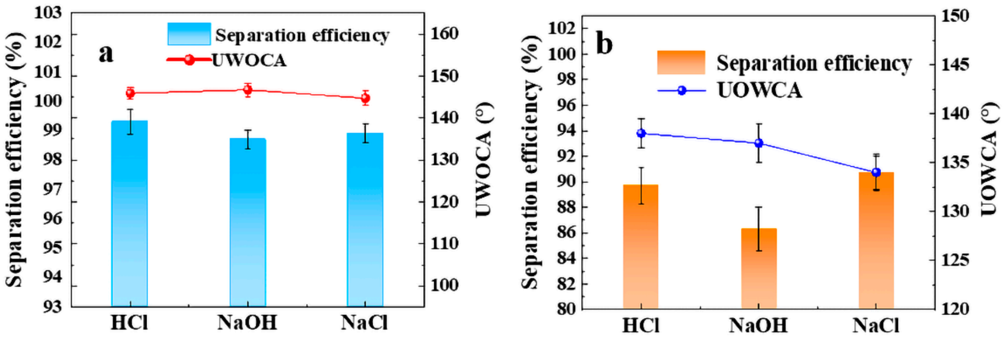

Figure S1. (a) Separation efficiency and the changes of UWOCA for O/W emulsion

(b) Separation efficiency and the changes of UOWCA for W/O emulsion

(Oil is n-octane)

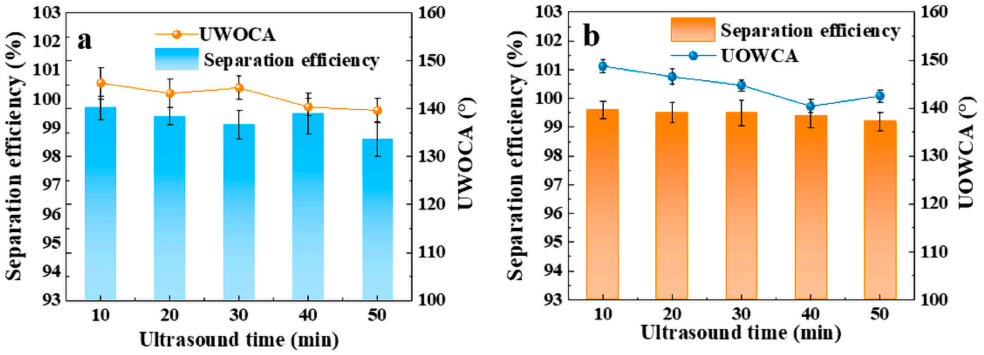

Figure S2. (a) Separation efficiency and the changes of UWOCA for O/W emulsion

(b) Separation efficiency and the changes of UOWCA for W/O emulsion

(Oil is n-octane)
